# The performance of BERT as data representation of text clustering

**DOI:** 10.1186/s40537-022-00564-9

**Published:** 2022-02-08

**Authors:** Alvin Subakti, Hendri Murfi, Nora Hariadi

**Affiliations:** Department of Mathematics, Universitas Indonesia, Depok, 16424 Indonesia

**Keywords:** Unsupervised learning, Text clustering, Representation learning, Deep learning, BERT

## Abstract

Text clustering is the task of grouping a set of texts so that text in the same group will be more similar than those from a different group. The process of grouping text manually requires a significant amount of time and labor. Therefore, automation utilizing machine learning is necessary. One of the most frequently used method to represent textual data is Term Frequency Inverse Document Frequency (TFIDF). However, TFIDF cannot consider the position and context of a word in a sentence. Bidirectional Encoder Representation from Transformers (BERT) model can produce text representation that incorporates the position and context of a word in a sentence. This research analyzed the performance of the BERT model as data representation for text. Moreover, various feature extraction and normalization methods are also applied for the data representation of the BERT model. To examine the performances of BERT, we use four clustering algorithms, i.e., k-means clustering, eigenspace-based fuzzy c-means, deep embedded clustering, and improved deep embedded clustering. Our simulations show that BERT outperforms TFIDF method in 28 out of 36 metrics. Furthermore, different feature extraction and normalization produced varied performances. The usage of these feature extraction and normalization must be altered depending on the text clustering algorithm used.

## Introduction

Information technology has an essential role in daily human activities and developing very quickly along with the times. The increasing availability of the internet supports the growth of information technology. One of the impacts of broader internet availability is increasing the digital text available online. Lockdown conditions during the Covid-19 pandemic also resulted in faster digital transformation.

Clustering is one of the tasks often used in digital text, i.e., grouping online news that enable us to find specific information based on the topic being discussed in the news. Grouping news can be done manually by analyzing the text in the news and determining the topics contained in the text. However, the large number of news available on the internet makes the manual grouping process inefficient. This is because grouping text data manually requires a lot of human resources and consumes a lot of time. Therefore, methods and algorithms that can be used to process and analyze text data automatically are necessary, one of which is machine learning. From a machine learning point of view, clustering is an unsupervised learning method utilizing unlabeled data [1]. Text data available on the internet generally do not have a label. Additionally, labeling text data also requires significant human resources. Due to these two reasons, the unsupervised learning method is suitable for determining groups in text data.

Text clustering is the process of grouping similar text from a set of texts and has several levels of granularity, namely document, paragraph, sentence, or phrase level. Text clustering has been in many fields such as book organization, corpus summarization, document classification [2], and topic detection [3]. To this date, various unsupervised learning algorithms have been implemented to perform text clustering. Some examples are k-means clustering (KM) [4], eigenspace-based fuzzy c-means (EFCM) [5], deep embedded clustering (DEC) [6], and improved deep embedded clustering (IDEC) [7].

One of the initial processes during text clustering is to represent text in the form of a numeric vector [8]. A model cannot directly process data in text form, so it must be transformed to a numeric format beforehand. Furthermore, the process of representing text can also help discover and learn patterns from the data. Representation learning is a method that automatically converts raw text data into its numeric representation. Representation learning methods commonly used are a bag of words methods such as Term Frequency-Inverse Document Frequency (TFIDF) [6, 7, 9, 10] and sequence of words methods such as word2vec and Bidirectional Encoder Representations from Transformers (BERT).

BERT is a pre-trained language model developed by Devlin et al. in 2018. The BERT model utilizes transformer model architecture to achieve State-of-The-Art (SOTA) performance for some Natural Language Processing (NLP) problems. BERT model can be used with two approaches which are feature-based approach and fine-tuning-based approach. In the feature-based process, BERT represents text data into fixed feature vectors using a pre-trained model. BERT can produce vector representations that take the position and context in a sentence into account [11]. Several studies have implemented the feature-based approach to obtain a text representation of the BERT model. Some of its applications are for toxic speech detection [12] and text classification [13, 14]. Most of the research conducted implements text data representation of BERT for solving supervised learning problems. However, research that focuses on implementing the representation method in unsupervised learning problems is still uncommon. This paper examines BERT as a text representation in unsupervised learning problems, namely text clustering. The simulations are performed on four standard text clustering methods, i.e., KM, EFCM, DEC, and IDEC. The performances of BERT are evaluated utilizing clustering accuracy (ACC), normalized mutual information (NMI), and adjusted rand index (ARI).

The simulation results showed that BERT outperforms TFIDF—the standard text representation—in 28 out of 36 metrics. Furthermore, different feature extraction and normalization produced varied performances. The usage of these feature extraction and normalization must be altered depending on the text clustering algorithm used. Moreover, we showed the reason behind high performance of BERT model as data representation with cluster visualization.

The rest of the paper is organized as follows: We present a literature review of the methods in the next section. Following this, we explain the methodologies used in this paper, and then we discuss the results of the simulations. Finally, we give a conclusion of this research in the last section.

## Method review

### Term frequency-inverse document frequency

TFIDF is a word representation method that can give constant weight to each word. In general, the representation of the TFIDF method implies the level of relevance of a word to a particular document. TFIDF considers two things, the frequency of words and the inverse of the frequency of occurrence of words in the document [15]. The numerical representation value of a word t in document d by TFIDF can be determined by using the following equation:

1 $$\begin{aligned} w_{t,d}=tf_{t,d}\times \log {\left( \frac{N}{df_t}\right) } , \end{aligned}$$

where $tf_{t,d}$ represents the frequency of *t* words in document *d*, *N* represents the number of documents, and $df_t$ represents the frequency of documents containing *t* words. The results of text data representation from TFIDF are used as input for various machine learning algorithms, one of which is text clustering algorithms.

### Bidirectional encoder representation from transformers

BERT is a pre-trained language model developed by Devlin et al. to improve the quality and efficiency of NLP solutions. The main architecture of BERT is the deep learning architecture of transformers encoder layers. BERT is composed of 12 layers of transformers encoder with each layer has a hidden size of 768, and the value of *h* in the multi-head self-attention layer is 12 [11]. This architecture is implemented in BERT to enumerate the significance of a word in a document based on its context.

The feature-based approach with BERT extracts fixed features from the pre-trained BERT model. This approach is also known as the contextualized word embedding. In contextualized word embedding, each word is mapped to a vector space, and words that have relatively similar meanings are relatively close in that vector space [16]. This approach has two advantages when compared to direct fine-tuning of the BERT model. The first advantage is that it can add a specific architecture specific to a problem since not all NLP problems can be solved using a transformer encoder architecture. The second advantage is that computational efficiency increased because the computationally expensive pre-computation representation is done only once, and the representation can be used in various experiments. Furthermore, since feature-based approach utilizes a pre-trained BERT model, it is scalable for use in large datasets.

The process of taking text representation using the feature-based approach of BERT is done by feeding a text input into BERT. The text input is tokenized using WordPiece Model before being fed into BERT. For a document containing n tokens, the text representation obtained is *n* numeric vectors with dimension 768. The output vector of all words in the document can be arranged into a matrix of size $n\times 768$.

### K-means clustering

K-means clustering is an algorithm that defines clusters as partitions of data [17]. K-Means Clustering algorithm aims to partition N data with *D* dimension into *D* clusters by minimizing an objective function [1]. For a *D*-dimensional data set $\{x_1,x_2,\ldots ,x_N\}$, the minimized objective function can be seen in the following equation:

2 $$\begin{aligned} J=\sum _{n=1}^N\sum _{k=1}^K r_{nk} ||x_n-\mu _k||^2 . \end{aligned}$$

The value of $r_{nk}\in \{0,1\}$ is the membership value of data $x_n$ in cluster *K*. The objective function *J* is the sum of the squares of the distances between each $x_n$ data point and each $\mu _k$ centroid. To minimize *J*, it is necessary to determine the appropriate values for $r_{nk}$ and $\mu _k$ by following an iterative procedure that goes through two stages, optimization of $r_{nk}$ followed by optimization of $\mu _k$. The objective function *J* can be minimized by assigning values of $\{r_nk\}$ and $\{\mu _k\}$ iteratively using these following equations respectively:

3 $$\begin{aligned} r_{nk}= & {} \left\{ \begin{array}{ll} 1, &{} \quad k=\arg {\min _k||x_n-\mu _k||^2} \\ 0, &{} \quad \text {others} \end{array} \right. \end{aligned}$$

4 $$\begin{aligned} \mu _k= & {} \dfrac{\sum _{n=1}^Nr_{nk}x_n}{\sum _{n=1}^Nr_{nk}}. \end{aligned}$$

### Eigenspace-based fuzzy c-means

Before explaining eigenspace-based fuzzy c-means, the fuzzy c-means (FCM) algorithm will be described first. FCM is a clustering algorithm where each data belongs to a cluster based on a degree of membership [18]. The concept of FCM is to determine the centroid of each cluster and the degree of membership of each data iteratively. This iteration is carried out until the value of the objective function is below the specified threshold, or the maximum number of iterations has been reached. The outputs of FCM are a set of centroids that minimizes the objective function and the degree of membership for each data in each existing cluster.

Suppose there is a set of data $\{x_1,x_2,\ldots ,x_N\}$ and a set of centroids ${\mu _1,\mu _2,\ldots ,\mu _K}$ both of which have dimension D. The sum of distances of each data to the center of the cluster can be expressed by the function J below [18]:

5 $$\begin{aligned} J=\sum _{n=1}^N\sum _{k=1}^K r_{nk}^m ||x_n-\mu _k||^2 . \end{aligned}$$

where $m>1$ is the degree of fuzziness and $r_{nk}^m$ indicates the degree of membership of the nth data in the *k*-th cluster with the additional known degree of fuzzy *m*. The value of $r_{nk}$ indicates how likely an observation can be part of a cluster.

The process of minimizing function *J* uses an iterative procedure that includes two stages, optimization of the value of $r_{nk}$ followed by optimization of the value of $\mu _k$. The optimal condition of the FCM algorithm is the values of $r_{nk}$ and $\mu _k$ that satisfies the equations below respectively:

6 $$\begin{aligned} r_{nk}= & {} \dfrac{1}{\sum _{j=1}^K\left( \dfrac{||x_n-\mu _k||}{||x_n-\mu _j||}\right) ^{\frac{2}{m-1}}}, \end{aligned}$$

7 $$\begin{aligned} \mu _k= & {} \dfrac{\sum _{n=1}^Nr_{nk}^mx_n}{\sum _{n=1}^Nr_{nk}^m}. \end{aligned}$$

FCM performs well on low-dimensional data but will fail to cluster on high-dimensional data. FCM will tend to produce the same centroid on high-dimensional data [19]. To overcome this problem, high-dimensional data needs to be transformed into low-dimensional data before FCM is carried out [5]; one of the methods is known as EFCM. Suppose there is a collection of data vectors arranged into a matrix $X=[x_1 x_2 \ldots x_n]$, where $x_i$ is the *i*-th data vector with dimension *m*. The data matrix *X* can be approximated using Truncated Singular Value Decomposition (TSVD) so that the decomposition of $X = \tilde{U}\tilde{\Sigma }\tilde{V}^T$ is obtained. Then the matrix X is represented by a lower-dimensional matrix $\tilde{\Sigma }\tilde{V}^T$ obtained from the decomposition. Then $\tilde{X}\approx \tilde{\Sigma }\tilde{V}^T$ will act as the result of dimension reduction of matrix X using TSVD and becomes an input for FCM.

### Deep embedded clustering

DEC is a clustering method where the optimization is done on the resulting cluster and optimizes the mapping parameters that map the data space to the latent space simultaneously. The simultaneous optimization process can improve cluster quality and feature space and reduce time complexity compared to methods that do not perform simultaneous optimization [6].

Suppose there is a set of *n* data $\{x_i\in X\}_{i=1}^n$ which will be grouped into *k* clusters. The cluster center represents each cluster $\mu _j$,$j=1,\ldots ,k$. In DEC, clustering is not carried out using data feature space *X*. The data is first transformed through a non-linear mapping $f_\theta : X \rightarrow Z$ where $\theta$ is the learned parameter and *Z* is the latent feature space. Parameterization of $f_\theta$ is carried out using a neural network structure. Dimension reduction in DEC uses a deep autoencoder structure and the DEC model is composed of two components: the encoder section and the clustering layer. DEC consists of two stages; the first stage is the initialization of the parameter in autoencoder $\theta$ and the centroid $\mu _j$. Meanwhile, the second stage is the simultaneous optimization of $\theta$ and $\mu _j$.

### Improved deep embedded clustering

DEC model has a weakness, where there is a distortion in the latent space due to the discarded decoder part when training the encoder using clustering loss. The IDEC method fixes these weaknesses by using the decoder section and attaching clustering loss to the latent space. This method is referred to as local structure preservation. In IDEC, autoencoder is studied by minimizing reconstruction loss. The initial learning process carried out at IDEC is the same as that carried out at DEC [7]. Suppose there is a set containing *n* data with dimension *d*, $x_i\in R^d$. The number of *K* clusters is known beforehand, and the cluster center is represented by $\mu _j\in R^d$. Non-linear mappings in the form of $f_W:x_i\rightarrow z_i$ and $g_w:z_i \rightarrow x_i'$ are also defined, where $z_i$ is the result of dimensionality reduction of $x_i$ and $x_i'$ is the reconstruction of $x_i$.

IDEC aims to find a good mapping $f_W$ such that the results of the reduced data ${z_i}_{i=1}^n$ are suitable for use in clustering problems. Two essential components to consider are autoencoder and clustering loss. Autoencoder is used to study the representation of unsupervised data so that the features obtained can preserve the local structure of the actual data. Clustering loss plays a role in manipulating the latent space so that the points that have been mapped in the latent space are more dispersed. Similar to the DEC, the learning process in IDEC is divided into two stages, the initialization stage and the optimization stage, by utilizing the preservation of local structures.

## Methodology

**Fig. 1:**
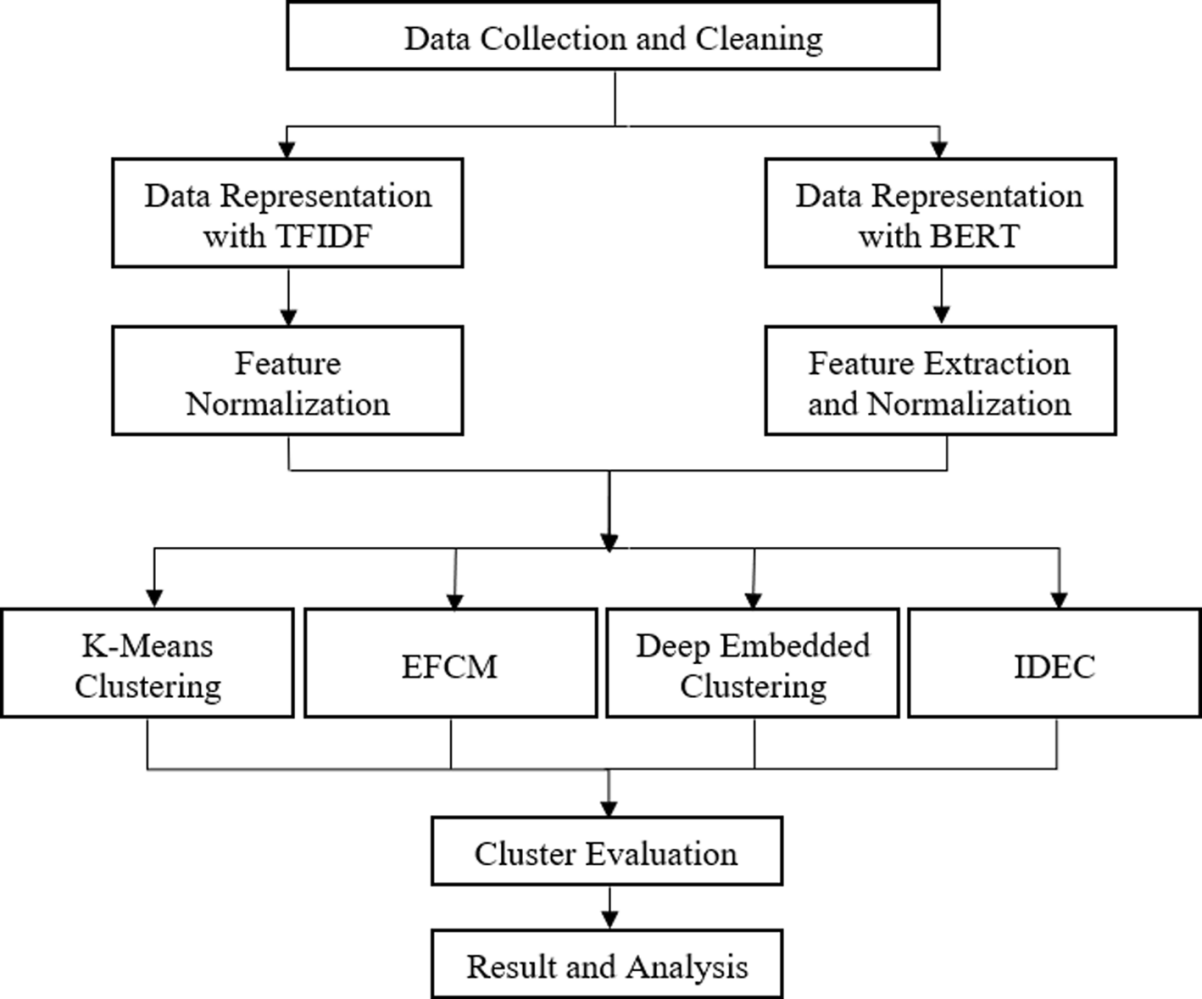
Flowchart of Simulation

In brief, Fig. 1 represents the flow of research methodology applied in this study.

It started with data collection, then continued with the extraction of text data representation from TFIDF and BERT. Feature normalization is applied after TFIDF, while BERT is followed by various feature extraction and normalization strategies.

Next, text clustering simulations are conducted by using four popular text clustering algorithms with different mechanisms. First, one of the popular traditional hard and soft clustering algorithms that is used in this research are K-Means Clustering [20] and Fuzzy C-Means (FCM) [18]. However in the case of data with high dimension, FCM did not perform well as the clusters will tend to have the same centroid [19]. Therefore FCM with dimensionality reduction is implemented, namely Eigenspace-based Fuzzy C-Means (EFCM) [5]. Then, the other two algorithms are some of the first deep learning based clustering algorithms that updates econder and cluster parameter simultaneously: DEC [6] and IDEC [7]. Finally the clusters produced will be evaluated and analyzed. The following paragraphs in this section will explain the methodologies stated above.

This study started with data collection. The data used are AG News, Yahoo! Answers, and R2 dataset that are explained in the next section. Text data representation is then extracted from the data. Two methods of extracting text representation used are TFIDF and BERT. Tokenization according to each technique of text data representation is carried out beforehand. After tokenization, the data is transformed using TFIDF and BERT. The representation of TFIDF is normalized to become input for DEC and IDEC, while the original representation is kept to become input for K-Means Clustering and EFCM. Text data representation obtained from BERT was also followed with several different extraction and normalization methods.

The fixed representation of text data from both methods is used for K-Means Clustering, EFCM, DEC, and IDEC. Evaluation of the resulting cluster is done by comparing the resulting group with ground-truth label data. The performance of the model was evaluated using clustering accuracy (ACC), normalized mutual information (NMI), and adjusted rand index (ARI). The performance between the representation from TFIDF and representation from BERT in text clustering is compared. Furthermore, the performance between text data representation from BERT with different extraction and feature normalization strategies are also compared.

### Data

Three popular datasets for text clustering are used [21, 22]: AG News, Yahoo! Answers, and R2 dataset that are originally collected by Zhang et al. [21]. However, due to the size of the datasets and time constraint, the reduced version of these datasets are adopted [22]. A brief description of the data used in this study can be seen in Table 1.

**Table 1:** Short description of the dataset

Dataset	Description	Number of class	Total number of data
AG News	Contains news titles and content from AG News media categorized by news topics	4	4000
Yahoo! Answers	Contains questions asked on Yahoo! Answers along with their answers which are categorized based on the topic of the question	10	10000
Reuters	Contains documents extracted from Reuters-21578, which is data containing news documents from the Reuters mass media in 1987	2	5859

Each class on AG news and Yahoo! Answers dataset consists of 1000 samples. This is because a small but balanced amount of data can produce a model similar to the original data [8]. A sampling of 1000 samples for each class reduces the computational load significantly without unduly affecting model performance. Meanwhile, the class distribution of the R2 is not uniform, with class 0 having 3724 data and class 1 having 2125 data.

The dataset used has been pre-processed in the previous research [8]. Some of the pre-processing conducted are combining all the lines in a document into one line. Secondly, any word that has a pattern of hashtags followed by a number is removed. The third pre-processing conducted is removing HTML or XML-related text and code from any documents. Lastly, whitespaces are removed, and repetitive punctuation marks are replaced with a single punctuation mark.

### Text rerpesentation

First, text representation from TFIDF is extracted. Tokenization with the help of the natural language toolkit (NLTK), where each word in a sentence is separated, is carried out beforehand. Next, the tokenized text data representation is taken by calculating the weight as described in Eq. 1. Then, to be used in DEC and IDEC models, normalization is applied to the text data representation generated by TFIDF. The representation is multiplied by the root of the feature dimension so that for an *i*-th text data representation vector, $x_i$ with the dimension *D*, we get $\frac{1}{D}||x_i||^2_2=1$

**Fig. 2:**
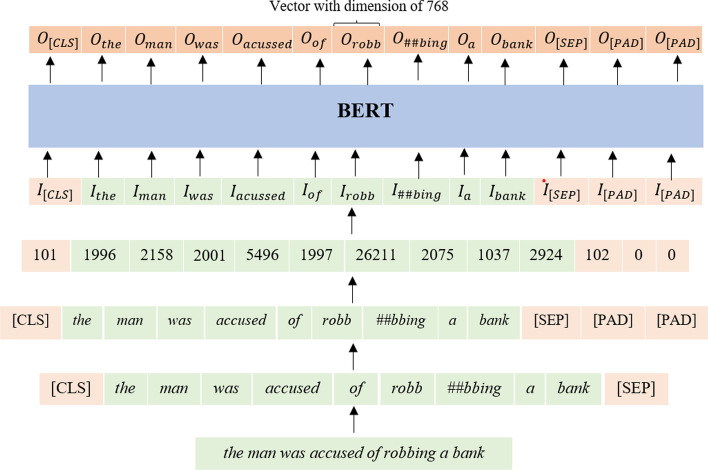
Illustration of text representation extraction from BERT

Next, text representation from BERT is extracted. The illustration of extracting text representation from BERT is shown in Fig. 2.

As shown in Fig. 2, before taking the representation from BERT, several additional pre-processing stages need to be conducted on the text data. These stages are tokenization, padding, and encoding. Tokenization for the BERT method is carried out using the WordPiece model, and the addition of special tokens [*CLS*] and [*SEP*] are added at the beginning and the end of the document.

Padding and truncating are performed to ensure each document in the data has the same length of tokens. The number of tokens for a document in this study is 25 tokens. Each document with less than 25 tokens will be padded with a special token [*PAD*] until the document length reaches 25 tokens. At the same time, documents that have more than 25 tokens will be truncated only up to the first 25 tokens and with the last token being the special token [*SEP*]. The next step is encoding, intending to map tokens into integers to process documents by BERT. The token encoding is performed by created a mapping with tokens from WordPiece model as keys and corresponding unique integers as values. The tokens in each document will be mapped to the corresponding integers so that another integer represents each token.

The BERT model used in this study is BERT-based uncased. BERT-based uncased is a BERT model that uses uncased data during pre-training. This model has 12 layers of transformer encoder, 768 hidden sizes, and 12 heads in the attention sub-layer. Text data representation is obtained by feeding forward the data that has been processed into the model as described previously. This study takes the text data representation from the output given from the second last transformer encoder layer (the 11th layer). The result obtained is a tensor of size (*n*, 25, 768), where n is the number of documents in the dataset, 25 is the number of tokens in the document, and 768 is the hidden size. Feature extraction and normalization strategies will be applied, resulting in a matrix of size (*n*, 768).

### Feature extraction and normalization strategies

A feature extraction strategy is necessary to convert high-dimensional representation from BERT into a fixed-sized feature vector with lower dimensions. Two feature extraction strategies were implemented, namely max pooling and mean pooling. Max pooling assumes that the highest value contains the most important features. Suppose that there are n tokens in a document and the *i*-th token has a vector representation as $h_i=[h_{i1},h_{i2},\ldots ,h_{id}]$ with dimension *d*. The max pooling strategy can be represented by the following equation [8]:

8 $$\begin{aligned} h[k]=\max _{1=1,\ldots ,n}h_{ik}, \end{aligned}$$

where *h*[*k*] is the *k*-th entry of the feature-extracted representation vector, *h*.

Mean pooling assumes that all contextual feature vectors can represent the entire text, and by taking the average value of these vectors, noise can be further reduced. The mean pooling strategy can be expressed with the equation below [8]:

9 $$\begin{aligned} h[k]=\dfrac{\sum _{i=1}^n h_{ik}}{n}. \end{aligned}$$

The vector representation output obtained from feature extraction will be the input in feature normalization.

Feature normalization is necessary to ensure that the fixed-sized vector representation has the characteristics of normality or stability. The four different normalization strategies implemented are identity normalization, standard normalization, layer normalization, and min–max normalization. Identity normalization is an identity function $f(h)=h$ and is used as a baseline for other normalization methods. For a feature vector $h_i$, standard normalization applies the function in the equation below [8]:

10 $$\begin{aligned} \overline{h_i}=\dfrac{h_i}{||h_i||}, \end{aligned}$$

where $\overline{h_i}$ is the normalized vector; it transforms vector representation into a vector with a norm of 1. The Euclidean distance between the two feature vectors will be equal to the cosine distance.

Layer normalization strategy can avoid covariate shift problems in the neural network training process [23]. The normalization layer applies the function in the equation below:

11 $$\begin{aligned} \overline{h_i} = \dfrac{h_i-\phi _i}{\sigma _i}, \end{aligned}$$

where $\phi _i$ and $\sigma _i$ are the mean and standard deviation of the feature vector $h_i$ respectively.

min–max normalization strategy is a normalization strategy that still preserves the initial distribution of the feature vectors. It applies the function in the equation below:

12 $$\begin{aligned} \overline{h_i} = \dfrac{h_i - \min _d{(h_{id})}}{\max _d{(h_{id})}-\min _d{(h_{id})}}. \end{aligned}$$

min–max normalization performs the transformation by scaling the feature vector $h_i$ which initially has a value interval of $[\min _d{(h_{i,d})}, \max _d{(h_{i,d})}]$ to [0, 1] [24].

### Text clustering simulation

The text clustering simulation in this study was done with 50 repetitions without making changes for each repetition. It aims to check the stability of the resulting text data representation. In this section, the hyperparameter settings used in the four text clustering models are described. For k-means clustering, the number of repetitions with different initial seeds in an algorithm is 10, the maximum number of iterations is 300, and the tolerance is $10^{-4}$. For EFCM, the number of components taken during TSVD is 5, the degree of fuzziness is 1.1, the tolerance is $10^{-4}$, and the maximum number of iterations is 200.

DEC and IDEC models have the same hyperparameter setting during the pre-training phase. The number of neurons in the autoencoder structure are $d-500-500-2000-5-2000-500-500-d$, with d being the dimension of the input data. The activation functions used in the encoder and decoder architecture are Rectified linear units, while linear activation function is used in the code and output layer. The proportion used as a validation during pre-training is 10%. The pre-training process implemented an early stopping mechanism during training with the maximum number of epochs is 500. The training process will be stopped early when its validation loss decreases with the patience value of 10. Furthermore, the best weights are kept during the training process and become the outcome when the training ends. Adaptive moment estimation (Adam) is used as the optimizer. During the optimization phase, it has the same value of $\alpha$, batch size, and update interval, which are 1, 256, and 30, respectively. Adam optimizer is also used during the optimization phase of both models. For the maximum error tolerance threshold, DEC used the threshold of $10^{-4}$, while IDEC used the value of $10^{-6}$.

## Result and discussion

### Evaluation metrics

The evaluation metric used is a metric that can measure the performance of the text clustering model using data that has a ground-truth label. In this research, three commonly used metrics are utilized to asses the quality of clusters, namely ACC [6–8, 22, 25], NMI [7, 8, 25], and ARI [8, 26, 27]. These three metrics evaluates cluster based on different considerations. Intuitively, ACC produced the best possible accuracy between ground-truth labels and clusters, NMI quantifies the amount of information about ground-truth labels given by clusters, and ARI measure the level of agreement between ground-truth labels and clusters. The details of these metrics are explained in this section.

ACC is an algorithm that searches for the best mapping between clusters obtained from the unsupervised algorithm used with ground-truth labels. The value of ACC can be determined by the following equation [28]:

13 $$\begin{aligned} ACC=\dfrac{\sum _{i=1}^n\delta (\alpha _1,map(l_i))}{n}, \end{aligned}$$

with $\alpha _i$ is the ground-truth label of the *i*-th data and $l_i$ is the label of the cluster obtained from the unsupervised algorithm. The function $\delta (x,y)$ is a function that will map to 1 if $x=y$ and map to 0 otherwise. The *map*(.) function will map an appropriate label for each cluster in a way such that it gives the best ACC value [6].

NMI is a normalized value of mutual information so that the value of mutual information, which was initially unlimited, becomes within the range of values [0, 1]. Let U be the ground-truth label, and V be the label from the unsupervised algorithm. The value of the NMI can be determined in the following equation [29]:

14 $$\begin{aligned} NMI(U,V) = \dfrac{MI(U,V)}{\sqrt{H(U)H(V)}}, \end{aligned}$$

*MI*(*U*, *V*) is a function of mutual information between clusters *V* and the ground-truth labels *U*. Normalization is done by dividing the value of the mutual information function by $\sqrt{(H(U)H(V)}$.

ARI determines its value based on the number of pairs of elements in the same subset and the number of pairs of elements in different subsets. ARI metric values can be determined using the following equation [26, 30]:

15 $$\begin{aligned} ARI=\dfrac{\sum _{i,j} \left( {\begin{array}{c}n_{ij}\\ 2\end{array}}\right) - \dfrac{\left[\sum _i\left( {\begin{array}{c}n_{i\cdot }\\ 2\end{array}}\right) \sum _j\left( {\begin{array}{c}n_{\cdot j}\\ 2\end{array}}\right) \right]}{\left( {\begin{array}{c}n\\ 2\end{array}}\right) } }{\frac{1}{2} \left[\sum _i\left( {\begin{array}{c}n_{i\cdot }\\ 2\end{array}}\right) +\sum _j\left( {\begin{array}{c}n_{\cdot j}\\ 2\end{array}}\right) \right] - \dfrac{\left[\sum _i\left( {\begin{array}{c}n_{i\cdot }\\ 2\end{array}}\right) \sum _j\left( {\begin{array}{c}n_{\cdot j}\\ 2\end{array}}\right) \right]}{\left( {\begin{array}{c}n\\ 2\end{array}}\right) }}, \end{aligned}$$

with n is the number of data, $n_{i\cdot }$ and $n_{j\cdot }$ represents the number of elements in each partition.

### Result

In this section, the results of simulations of the four models previously described are shown and analyzed. The results of clustering in each dataset are evaluated using ACC, NMI, and ARI, such that there are a total of 36 metrics considered from 3 datasets and 4 clustering algorithms. The measured metric is the quality of the clusters generated by text data representation from TFIDF and BERT on KM, EFCM, DEC, and IDEC. This study compares the performance between different text data representations rather than finding the overall best performing method. Tables 2, 3, and 4 summarize different text data representation metrics on different text clustering models using the AG News, Yahoo! Answers, and R2 datasets, respectively.

**Table 2:** Cluster evaluation on AG News dataset

Method	AG news		
	ACC	NMI	ARI
TFIDF + KM	0.5019 ± 0.0718	0.2559 ± 0.0802	0.2552 ± 0.0803
BERT + Max + I + KM	0.7674 ± 0.0018	0.4872 ± 0.0021	0.4868 ± 0.0021
BERT + Max + LN + KM	0.7913 ± 0.0040	0.5199 ± 0.0050	0.5195 ± 0.0050
BERT + Max + N + KM	0.7858 ± 0.0017	0.5136 ± 0.0025	0.5132 ± 0.0025
BERT + Max + MM + KM	0.4408 ± 0.0012	0.1986 ± 0.0014	0.1979 ± 0.0014
BERT + Mean + I + KM	0.6491 ± 0.0016	0.4196 ± 0.0010	0.4191 ± 0.0010
**BERT + Mean + LN + KM**	**0.6468** ± **0.0036**	**0.4152** ± **0.0018**	**0.4148** ± **0.0018**
BERT + Mean + N + KM	0.6467 ± 0.0033	0.4151 ± 0.0017	0.4146 ± 0.0017
BERT + Mean + MM + KM	0.3208 ± 0.0051	0.0441 ± 0.0008	0.0432 ± 0.0008
TFIDF + EFCM	0.5788 ± 0.03197	0.2979 ± 0.0309	0.2973 ± 0.0309
BERT + Max + I + EFCM	0.7561 ± 0.0004	0.4731 ± 0.0006	0.4726 ± 0.0006
**BERT + Max + LN + EFCM**	**0.778** ± **0.0002**	**0.4976** ± **0.0004**	**0.4972** ± **0.0004**
BERT + Max + N + EFCM	0.7642 ± 0.0003	0.4841 ± 0.0004	0.4837 ± 0.0004
BERT + Max + MM + EFCM	0.4439 ± 0.0085	0.1997 ± 0.0100	0.1991 ± 0.0100
BERT + Mean + I + EFCM	0.6449 ± 0.0003	0.4086 ± 0.0002	0.4081 ± 0.0002
BERT + Mean + LN + EFCM	0.6423 ± 0.0003	0.4088 ± 0.0003	0.4083 ± 0.0003
BERT + Mean + N + EFCM	0.6425 ± 0.0003	0.4089 ± 0.0003	0.4084 ± 0.0003
BERT + Mean + MM + EFCM	0.3067 ± 0.0037	0.0429 ± 0.0003	0.0421 ± 0.0003
TFIDF + DEC	0.7211 ± 0.0250	0.3861 ± 0.0265	0.4139 ± 0.0338
BERT + Max + I + DEC	0.2539 ± 0.0274	0.0037 ± 0.0259	0.003 ± 0.0210
BERT + Max + LN + DEC	0.7677 ± 0.0436	0.4878 ± 0.0344	0.513 ± 0.0483
BERT + Max + N + DEC	0.2585 ± 0.0326	0.004 ± 0.0179	0.0033 ± 0.0162
BERT + Max + MM + DEC	0.3529 ± 0.1505	0.0817 ± 0.1476	0.0798 ± 0.1461
BERT + Mean + I + DEC	0.7719 ± 0.0506	0.5055 ± 0.0363	0.5304 ± 0.0518
BERT + Mean + LN + DEC	0.7653 ± 0.0550	0.4987 ± 0.0426	0.5206 ± 0.0579
**BERT + Mean + N + DEC**	**0.8038** ± **0.0325**	**0.538** ± **0.0210**	**0.5707** ± **0.0296**
BERT + Mean + MM + DEC	0.25 ± 0	0.0004 ± 0.0018	0 ± 0
TFIDF + IDEC	0.7453 ± 0.0243	0.4251 ± 0.0244	0.4571 ± 0.0315
BERT + Max + I + IDEC	0.376 ± 0.1413	0.1467 ± 0.1565	0.1253 ± 0.1457
BERT + Max + LN + IDEC	0.7819 ± 0.0411	0.5131 ± 0.0294	0.5394 ± 0.0428
BERT + Max + N + IDEC	0.3618 ± 0.1478	0.1163 ± 0.1511	0.1072 ± 0.1408
BERT + Max + MM + IDEC	0.4077 ± 0.111	0.1157 ± 0.1269	0.1093 ± 0.1222
BERT + Mean + I + IDEC	0.7836 ± 0.0509	0.5296 ± 0.0353	0.5544 ± 0.0511
BERT + Mean + LN + IDEC	0.782 ± 0.0541	0.5297 ± 0.0398	0.5524 ± 0.0548
**BERT + Mean + N + IDEC**	**0.8019** ± **0.0330**	**0.5383** ± **0.0217**	**0.5688** ± **0.0312**
BERT + Mean + MM + IDEC	0.2616 ± 0.0208	0.0165 ± 0.0184	0.0026 ± 0.0063

The feature extraction and normalization strategies are abbreviated into Max for max pooling, Mean for mean pooling, I for identity normalization, LN for layer normalization, N for standard normalization, and MM for min–max normalization. The deviations denote the standard deviation of the metric from 50 repetitions. The values in bold denote the highest value in every metric in each text clustering algorithm. While the methods in bold, if there are any, is the best performing method in each text clustering algorithm

Table 2 shows the evaluation of the metric of different text data representations on different text clustering on the AG News dataset. In all four models, text data representation from BERT can outperform representation from TFIDF. As shown on Table 2, text data representation from BERT followed by max-pooling and layer normalization performs best in KM and EFCM model. Additionally, text data representation from BERT followed by mean pooling and standard normalization performs best in DEC and IDEC models.

**Table 3:** Cluster evaluation on Yahoo! Answers dataset

Method	AG news		
	ACC	NMI	ARI
TFIDF + KM	0.3568 ± 0.0059	0.2135 ± 0.0067	0.2121 ± 0.0068
BERT + Max + I + KM	0.3018 ± 0.0131	0.1495 ± 0.0095	0.1479 ± 0.0095
BERT + Max + LN + KM	0.3285 ± 0.0140	0.1797 ± 0.0132	0.1782 ± 0.0132
BERT + Max + N + KM	0.3229 ± 0.0145	0.1739 ± 0.0137	0.1724 ± 0.0137
BERT + Max + MM + KM	0.226 ± 0.0058	0.088 ± 0.0057	0.0864 ± 0.0057
BERT + Mean + I + KM	0.357 ± 0.0079	0.2134 ± 0.0077	0.212 ± 0.0077
**BERT + Mean + LN + KM**	**0.3741** ± **0.0057**	**0.2302** ± **0.0055**	**0.2288** ± **0.0055**
BERT + Mean + N + KM	0.373 ± 0.0071	0.2286 ± 0.0066	0.2273 ± 0.0066
BERT + Mean + MM + KM	0.1718 ± 0.0066	0.0511 ± 0.0050	0.0493 ± 0.0050
TFIDF + EFCM	0.2482 ± 0.0081	0.1177 ± 0.0018	0.1161 ± 0.0018
BERT + Max + I + EFCM	0.2484 ± 0.0070	0.122 ± 0.0029	0.1204 ± 0.0029
**BERT + Max + LN + EFCM**	0.2454 ± 0.0069	**0.1302** ± **0.0015**	**0.1287** ± **0.0015**
BERT + Max + N + EFCM	0.2374 ± 0.0051	0.1255 ± 0.0014	0.1239 ± 0.0014
BERT + Max + MM + EFCM	0.2043 ± 0.0098	0.0706 ± 0.0044	0.0689 ± 0.0044
BERT + Mean + I + EFCM	0.2486 ± 0.0077	0.1174 ± 0.0016	0.1158 ± 0.0016
BERT + Mean + LN + EFCM	0.2522 ± 0.0037	0.1253 ± 0.0012	0.1237 ± 0.0012
**BERT + Mean + N + EFCM**	**0.2523** ± **0.0032**	0.1252 ± 0.0010	0.1236 ± 0.0010
BERT + Mean + MM + EFCM	0.1632 ± 0.0066	0.0415 ± 0.0019	0.0397 ± 0.0019
TFIDF + DEC	0.4024 ± 0.0282	0.2176 ± 0.0154	0.1621 ± 0.0202
BERT + Max + I + DEC	0.1061 ± 0.0143	0.003 ± 0.0074	0.0015 ± 0.0038
BERT + Max + LN + DEC	0.3969 ± 0.0186	0.2301 ± 0.0143	0.1761 ± 0.0133
BERT + Max + N + DEC	0.1 ± 0	0 ± 0	0 ± 0
BERT + Max + MM + DEC	0.1713 ± 0.0708	0.0539 ± 0.0638	0.0312 ± 0.0397
BERT + Mean + I + DEC	0.4661 ± 0.0282	0.286 ± 0.0121	0.2317 ± 0.0193
**BERT + Mean + LN + DEC**	**0.4754** ± **0.0266**	**0.2907** ± **0.0119**	**0.2339** ± **0.0172**
BERT + Mean + N + DEC	0.427 ± 0.0292	0.2613 ± 0.013	0.1992 ± 0.0172
BERT + Mean + MM + DEC	0.1 ± 0	0.0001 ± 0	0 ± 0
TFIDF + IDEC	0.3975 ± 0.0235	0.2243 ± 0.0109	0.1474 ± 0.0111
BERT + Max + I + IDEC	0.1326 ± 0.0354	0.0225 ± 0.0241	0.0135 ± 0.0158
BERT + Max + LN + IDEC	0.4058 ± 0.0182	0.2394 ± 0.0129	0.1881 ± 0.0131
BERT + Max + N + IDEC	0.1242 ± 0.0342	0.0193 ± 0.0275	0.0097 ± 0.0144
BERT + Max + MM + IDEC	0.1694 ± 0.0511	0.0504 ± 0.0497	0.0278 ± 0.0301
BERT + Mean + I + IDEC	0.477 ± 0.0294	0.2988 ± 0.0126	0.2445 ± 0.0199
**BERT + Mean + LN + IDEC**	**0.487** ± **0.0258**	**0.3019** ± **0.0118**	**0.247** ± **0.0167**
BERT + Mean + N + IDEC	0.4308 ± 0.0303	0.2687 ± 0.0134	0.2078 ± 0.0170
BERT + Mean + MM + IDEC	0.1015 ± 0.0029	0.0081 ± 0.005	7E-05 ± 0.0004

The feature extraction and normalization strategies are abbreviated into Max for max pooling, Mean for mean pooling, I for identity normalization, LN for layer normalization, N for standard normalization, and MM for min–max normalization. The deviations denote the standard deviation of the metric from 50 repetitions. The values in bold denote the highest value in every metric in each text clustering algorithm. While the method in bold, if there are any, is the best performing method in each text clustering algorithm.

The clusters result on Yahoo! Answers dataset shown in Table 3 is aligned with the previous results in Table 2, where text data representation from BERT can outperform the representation from TFIDF. In DEC and IDEC model, the best performing representation is the same as in Table 2. In addition, this representation also performs best compared to other representations in KM. In EFCM, there are two best performing representation based on different metrics. The highest value of ACC was shown in text representation from BERT, followed by mean pooling and layer normalization. On the other hand, the highest value of NMI and ARI metrics was shown in text representation from BERT followed by max-pooling and layer normalization.

**Table 4:** Cluster evaluation on R2 dataset

Method	AG news		
	ACC	NMI	ARI
TFIDF + KM	0.8471 ± 0	0.5034 ± 0	0.5033 ± 0
BERT + Max + I + KM	0.8457 ± 0	0.5025 ± 0	0.5024 ± 0
BERT + Max + LN + KM	0.8472 ± 0	**0.5052** ± **0**	**0.5052** ± **0**
BERT + Max + N + KM	0.8469 ± 0	0.4985 ± 0.0015	0.4984 ± 0.0015
BERT + Max + MM + KM	0.8495 ± 0	0.4942 ± 0	0.4941 ± 0
BERT + Mean + I + KM	0.8471 ± 0	0.5034 ± 0	0.5033 ± 0
BERT + Mean + LN + KM	**0.8507** ± **0**	0.5036 ± 0	0.5035 ± 0
BERT + Mean + N + KM	**0.8507** ± **0**	0.5036 ± 0	0.5035 ± 0
BERT + Mean + MM + KM	0.6624 ± 0.0002	0.0822 ± 0.0003	0.0821 ± 0.0003
TFIDF + EFCM	0.8476 ± 0	**0.5043** ± **0**	**0.5042** ± **0**
BERT + Max + I + EFCM	0.8462 ± 0	0.5034 ± 0	0.5033 ± 0
BERT + Max + LN + EFCM	0.8474 ± 0	0.504 ± 0	0.5039 ± 0
BERT + Max + N + EFCM	0.8479 ± 0	0.4964 ± 0	0.4964 ± 0
BERT + Max + MM + EFCM	0.8498 ± 0	0.4957 ± 0	0.4957 ± 0
BERT + Mean + I + EFCM	0.8476 ± 0	**0.5043** ± **0**	**0.5042** ± **0**
BERT + Mean + LN + EFCM	**0.8505** ± **0**	0.5 ± 0	0.4999 ± 0
BERT + Mean + N + EFCM	**0.8505** ± **0**	0.5 ± 0	0.4999 ± 0
BERT + Mean + MM + EFCM	0.6636 ± 0	0.0827 ± 0	0.0826 ± 0
**TFIDF + DEC**	**0.859** ± **0.0100**	**0.5064** ± **0.0205**	**0.5158** ± **0.0288**
BERT + Max + I + DEC	0.793 ± 0.0794	0.386 ± 0.1525	0.3545 ± 0.1835
BERT + Max + LN + DEC	0.8409 ± 0.0188	0.4827 ± 0.0308	0.466 ± 0.0480
BERT + Max + N + DEC	0.8474 ± 0.0033	0.4996 ± 0.0078	0.4825 ± 0.0092
BERT + Max + MM + DEC	0.7816 ± 0.0590	0.3727 ± 0.1332	0.3269 ± 0.1348
BERT + Mean + I + DEC	0.8497 ± 0.0025	0.504 ± 0.0068	0.4891 ± 0.0070
BERT + Mean + LN + DEC	0.8494 ± 0.0017	0.5035 ± 0.0059	0.4882 ± 0.0047
BERT + Mean + N + DEC	0.8533 ± 0.0045	0.4996 ± 0.0059	0.4993 ± 0.0128
BERT + Mean + MM + DEC	0.6373 ± 0	0.00002 ± 0.0001	0.00001 ± 0.00009
**TFIDF + IDEC**	**0.8654** ± **0.0116**	**0.5213** ± **0.0252**	**0.5345** ± **0.0342**
BERT + Max + I + IDEC	0.8095 ± 0.0616	0.4303 ± 0.1373	0.3917 ± 0.1442
BERT + Max + LN + IDEC	0.8401 ± 0.0228	0.485 ± 0.0349	0.4643 ± 0.0572
BERT + Max + N + IDEC	0.8428 ± 0.0297	0.4889 ± 0.0704	0.4718 ± 0.0686
BERT + Max + MM + IDEC	0.7815 ± 0.0588	0.3623 ± 0.1399	0.3255 ± 0.1366
BERT + Mean + I + IDEC	0.8494 ± 0.0011	0.507 ± 0.0049	0.4881 ± 0.0032
BERT + Mean + LN + IDEC	0.8494 ± 0.0007	0.5045 ± 0.0048	0.4884 ± 0.0021
BERT + Mean + N + IDEC	0.8518 ± 0.0038	0.4952 ± 0.0068	0.4951 ± 0.0108
BERT + Mean + MM + IDEC	0.6374 ± 0.0002	0.0007 ± 0.0014	0.0004 ± 0.0007

The feature extraction and normalization strategies are abbreviated into Max for max pooling, Mean for mean pooling, I for identity normalization, LN for layer normalization, N for standard normalization, and MM for min–max normalization.The deviations denote the standard deviation of the metric from 50 repetitions. The values in bold denote the highest value in every metric in each text clustering algorithm. While the method in bold, if there are any, is the best performing method in each text clustering algorithm

The results shown in Table 4 are relatively different from the previous two tables. Text data representations from BERT could not outperform TFIDF, when used in both DEC and IDEC models. While in KM and EFCM, representation from BERT followed by mean pooling and layer normalization or standard normalization have the best performance with TFIDF still have a competitive performance.

Overall, the results obtained that the text data representation using BERT outperformed text data representation using TFIDF on 28 out of 36 metrics. In addition, in the remaining 8 out of 36 metrics, the performance of text data representation from BERT can still compete with TFIDF. The representation of text data from BERT on average decreased by 1.117% on the ACC metric, 0.953% on the NMI metric, and 3.52% on the ARI metrics. This decrease is not significant compared to the increase experienced on the other 28 metrics.

One of the reasons for the high performance of text data representation from BERT is because BERT can produce a representation that positions similar texts closer. On top of that, the Euclidean distance between two text features can represent the semantic relationship between the two. Visualization of text data representation was carried out using the TFIDF method and the BERT method using t-SNE. Two situations will be visualized, the first situation is when representation from BERT outperforms TFIDF, and the second one is the other way around. Henceforth, the text data representation used in the t-SNE visualization is TFIDF with normalization and BERT model with mean pooling and standard normalization.

**Fig. 3:**
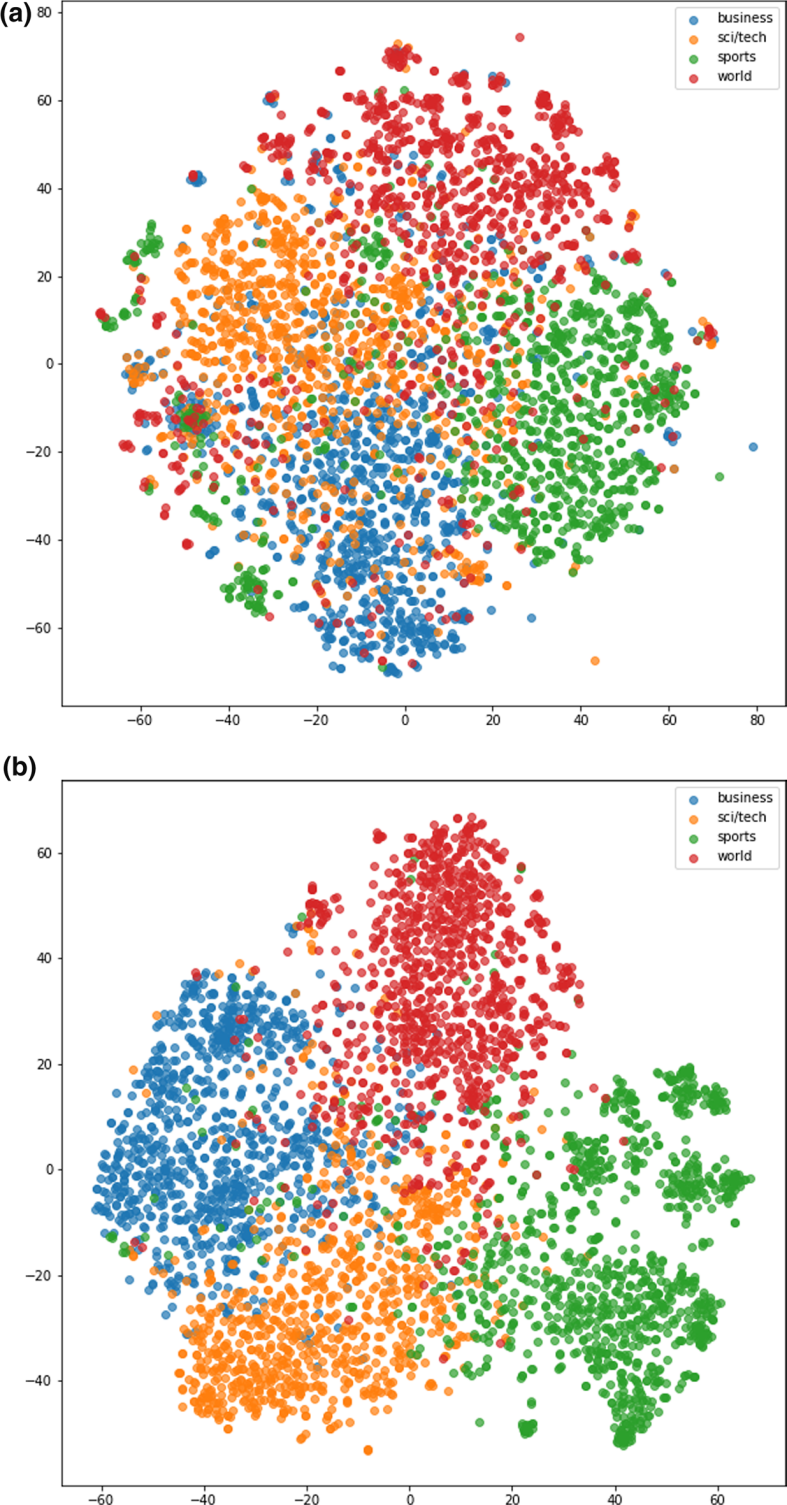
t-SNE visualization of AG News ground truth label with text data representation from (**a**) TFIDF (**b**) BERT

First, we show a visualization for the situation when text data representation from BERT outperforms TFIDF. The ground-truth label visualization of AG news using text data representation can be seen in Fig. 3. It can be observed from Fig. 3a that the groups between different classes are not well differentiated. The world-class is marked in red, the sci/tech class is marked in orange, and the business class is marked in blue. This behavior is not shown in Fig. 3b, where the clusters between the four classes can be distinguished. These align with the performance of the text clustering model on AG news which is shown in Table 2, where text data representation from BERT followed by mean normalization and standard normalization is better than the TFIDF.

**Fig. 4:**
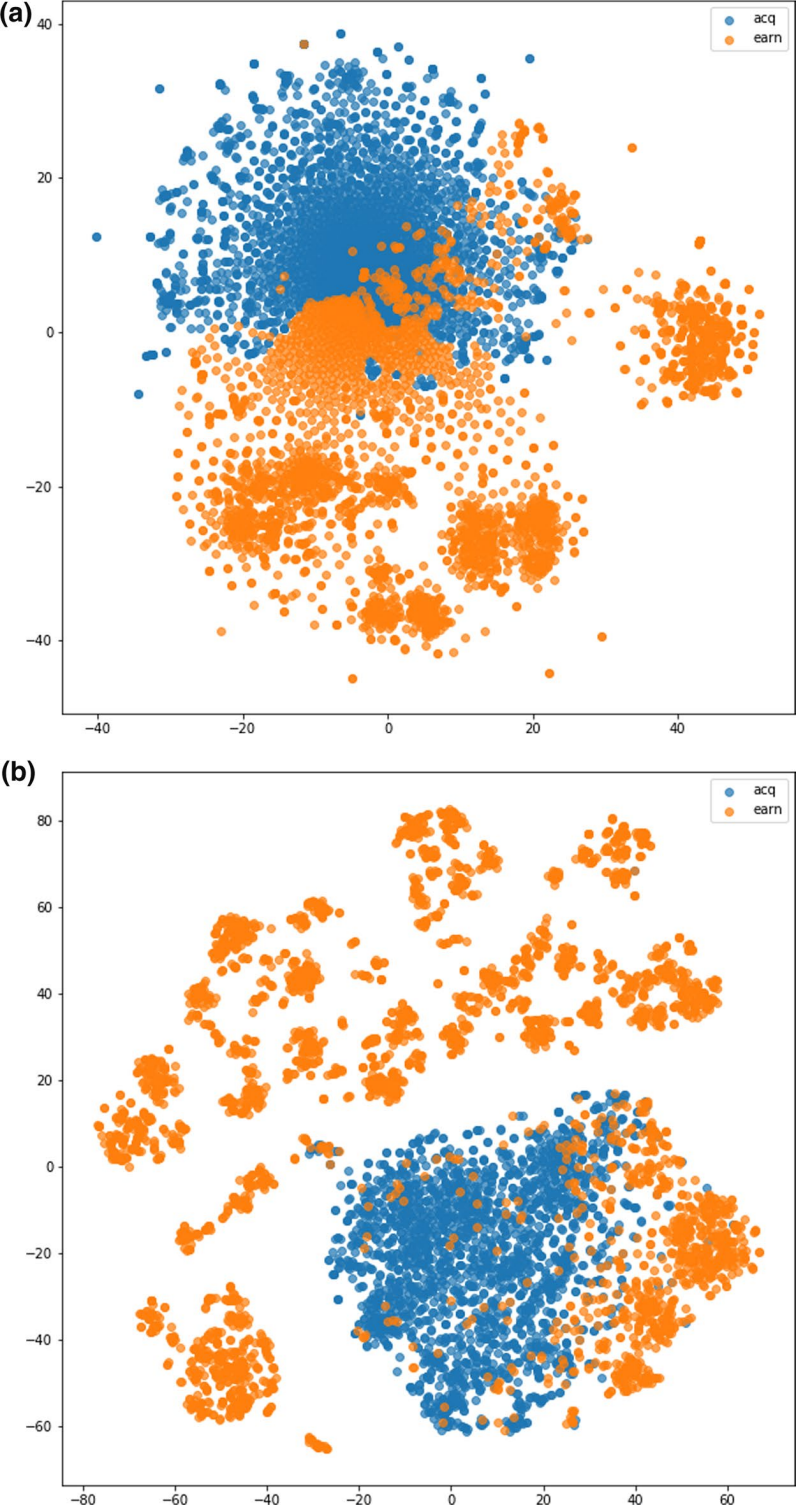
t-SNE visualization of R2 ground truth label with text data representation from (**a**) TFIDF (**a**) BERT

Next, a visualization for the situation when text data representation from TFIDF outperforms BERT on R2 data is shown in Fig. 4. It can be observed from Fig. 4a that there is an area in the middle where members of the acq and earn class is mixed. Meanwhile, it can also be observed from Fig. 4b that a proportion of earn class in the lower right is apart from other earn class elements. The separated observations of the earn class also has a shorter distance to the acq class. This results in the performance of the two text data representations being quite similar. The performance of the two representations can be seen in Table 4. The text data representation from the TFIDF method followed by normalization has slightly better performance than BERT, followed by mean pooling and standard normalization.

To analyze different extraction and normalization strategies used in text data representation from BERT, the metrics will be divided into two based on the text clustering model used. The division is composed of KM with EFCM and the DEC with IDEC. The total of metrics reviewed in each division is 18 metrics.

When clustering using KM and EFCM, the combination of max pooling and layer normalization has the best performance on 10 out of 18 metrics. The rest 8 out of 18 metrics still have a competitive result compared with metrics produced by other extraction and normalization methods combinations. On the other hand, when clustering with DEC and IDEC models, the combination of mean pooling and standard normalization has the best performance on 10 out of 18 metrics. The other 8 out of 18 metrics still have similar results with the extracted and normalization methods, although it is not the best performance. These results show that the best feature extraction and normalization differ depending on the text clustering model used.

Another insight obtained is that the lowest performance for all 36 metrics occurred when using text data representation from BERT with mean pooling followed by min–max normalization. In particular, the lowest performance can also outperform the TFIDF method, which is the baseline in this study.

**Fig. 5:**
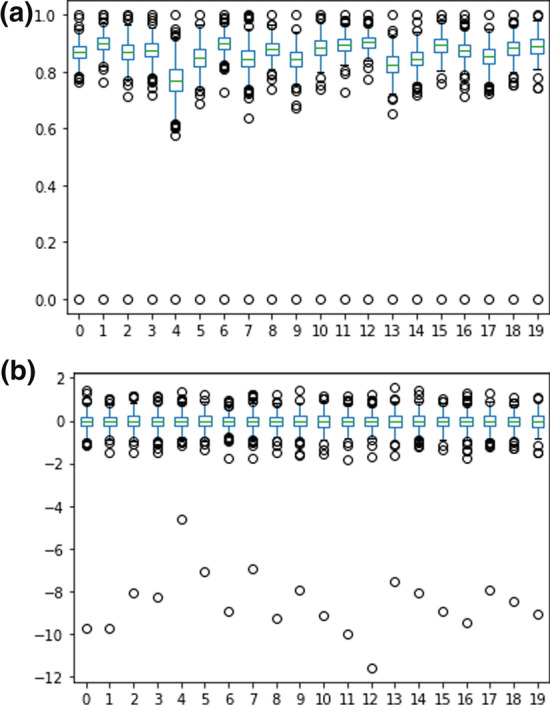
Boxplot of the first 20 AG News data representation from BERT + Mean (**a**) before min–max normalization (**b**) after min–max normalization

One of the causes of this poor performance is the min–max normalization process which is prone to outliers. Suppose some values are much higher or much lower than the other values in the text data representation. In that case, the results from min–max normalization will still have these outliers and the same distribution as before. This aligns with the condition of text data representation using the BERT method followed by mean pooling. This text data representation has a much smaller value compared to the other values in each document. This can be seen in the box diagram of Fig. 5.

In addition, the outlier value shown in Fig. 5 is an element with the same position in each vector of AG news data representation. By transforming the outlier value to 0, every AG news representation vector element with that position will also be 0. This eliminates information that may be contained in the element at that position.

## Conclusion

In this paper, we aimed to analyze the performance of BERT as a text data representation. The performance of representation was evaluated by becoming an input for four text clustering algorithms, namely KM, EFCM, IDEC, and IDEC. Then, the clusters obtained from the algorithms were evaluated using ACC, NMI, and ARI metrics. Based on the results, BERT was able to outperform TFIDF, to represent text data in text clustering on 28 of the 36 investigated metrics. This is due to the performance of text data representation from BERT which produce a representation that positions similar texts closer.

Furthermore, results obtained also shows that feature extraction and feature normalization methods applied to representation from BERT produce different performances depending on the text clustering model used. Representation from BERT followed with max-pooling, and layer normalization outperformed other method on 10 of the 18 metrics investigated in the KM and EFCM models. Then, Representation from BERT followed with mean pooling and standard normalization outperformed other method on 10 of the 18 metrics investigated in the DEC and IDEC models. In future work, development of deep learning based clustering algorithms can be oriented more toward representation utilizing BERT model rather than TFIDF.

## Data Availability

The datasets used and/or analyzed during the current study are available from the corresponding author on reasonable request.

## Abbreviations

ACC: Clustering accuracy
ARI: Adjusted rand index
BERT: Bidirectional Encoder Representations from Transformers
EFCM: Eigenspace-based fuzzy c-means
DEC: Deep embedded clustering
FCM: Fuzzy c-means
I: Identity normalization
IDEC: Improved deep embedded clustering
KM: K-means clustering
LN: Layer normalization
MM: Min–max normalization
N: Standard Normalization
NLP: Natural Language Processing
NLTK: Natural language toolkit
NMI: Normalized mutual information
SOTA: State-of-the-art
TFIDF: Term Frequency-Inverse Document Frequency
TSVD: Truncated Singular Value Decomposition
